# Towards quantifying microbial dispersal in the environment

**DOI:** 10.1111/1462-2920.16270

**Published:** 2022-11-04

**Authors:** Kristin M. Barbour, Alberto Barrón‐Sandoval, Kendra E. Walters, Jennifer B. H. Martiny

**Affiliations:** ^1^ Department of Ecology and Evolutionary Biology University of California‐Irvine Irvine California USA; ^2^ Department of Biology Reed College Portland Oregon USA

Dispersal, the movement of individuals across space, has long been recognized as a fundamental process influencing the assembly of plants and animals (Ronce, 2007; Vellend, 2010). In contrast, the importance of dispersal for microbial communities remains contentious. For the past two decades, microbiologists have intensely debated the tenet ‘everything is everywhere, but the environment selects’ (Baas‐Becking, 1934). Baas‐Becking's statement surmises that microorganisms do not experience barriers to movement—, that is, they are not dispersal limited. However, over the last two decades, a large and growing body of evidence indicates that microbes are restricted in dispersal (see below). Despite these advances, we still lack basic knowledge—the who, how, and how fast—of microbial dispersal in the environment. Efforts to explicitly characterize microbial dispersal in the environment are necessary as the dispersal process is foundational to the composition and resilience of microbial communities (Nemergut et al., 2013; Shade et al., 2012), as well as to pathogen spread (Cevallos‐Cevallos et al., 2012) and microbiome engineering (Albright et al., 2021; Rocca et al., 2021).

Before looking forward, we discuss our current understanding of microbial dispersal. While an exhaustive review is beyond the scope of this article (see Chaudhary et al., 2022; Choudoir & DeAngelis 2022; Custer et al., 2022), we summarize key evidence for dispersal limitation and its potential effects on microbial communities.

## CURRENT EVIDENCE FOR DISPERSAL LIMITATION AND ITS EFFECTS ON MICROBIAL COMMUNITIES

Our current understanding of microbial dispersal is primarily derived from inference‐based studies and dispersal manipulation experiments. The inference‐based evidence is based on spatial biogeographic patterns of microbial diversity. For instance, at the community scale, it is now well established that microbial communities generally follow a distance‐decay pattern (Franklin & Mills, 2003; Hillebrand et al., 2001; Reche et al., 2005), where compositional similarity decreases as spatial distance increases between two communities. When this pattern holds even after controlling for spatial autocorrelation of the environment, it suggests that dispersal is limited (Martiny et al., 2006). At finer genetic scales, microbial taxa also exhibit isolation‐by‐distance patterns, where the genetic differentiation of bacterial populations increases with greater geographic distance between them (Cho & Tiedje, 2000; Papke et al., 2003; Whitaker et al., 2003). If dispersal (and therefore, gene flow) is restricted, then chance events within populations can contribute to genetic divergence. Complementary to decay patterns, Andam et al. (2016) found that the genetic diversity of *Streptomyces* increases with decreasing latitude, a pattern consistent with recent glacial retreat. Thus, modern mixing of *Streptomyces* populations does not appear to have erased historical legacies of thousands of years (Martiny, 2016).

Although these observations are consistent with the idea of dispersal limitation, they cannot rule out the possibility that unmeasured environmental (biotic and abiotic) parameters are solely responsible for the patterns. That said, the large body of studies coming to the same conclusion is powerful evidence that microbes are dispersal limited (Hanson et al., 2012).

More recently, experiments have moved beyond inference‐based patterns to directly manipulate microbial dispersal in controlled systems. In the laboratory, ecological dynamics of simplified microbial communities (often an artificial mixture of isolates) are assessed in microcosms with and without the addition of cells from an assembled source pool. These studies find that dispersal can affect microbial evolution, community assembly, and community functioning. Further, the effects of dispersal vary based on a range of factors including the size, timing, and composition of the dispersing community (Jones et al., 2017; Svoboda et al., 2018; van Elsas et al., 2012; Zha et al., 2016). In addition, field experiments that exclude all immigration into a community are a powerful method for demonstrating the potential impact of dispersal in the field. Using this method, Albright and Martiny (2018) showed that excluding all dispersal altered the abundance, diversity, and composition of surface leaf litter communities.

While these experiments underscore the potential importance of dispersal for microbial communities, their applicability to natural communities remains unclear. In the laboratory, the rates and composition of the communities used remain largely arbitrary because of the dearth of quantitative information on microbial dispersal in natural environments. In the field, exclusion experiments provide an unrealistic scenario that is difficult to extrapolate to likely changes in dispersal.

## FUTURE AVENUES FOR MICROBIAL DISPERSAL RESEARCH

Combined, the evidence above overwhelmingly suggests that microbial dispersal is indeed limited and to some extent, influences microbial communities in the environment. We submit that the future of microbial dispersal research is to move beyond questions that address the potential consequences of dispersal to those that assess the actual role of dispersal in intact, environmental communities. Specifically, we propose prioritizing three questions: (1) What rate are microbes moving? (2) Where are they coming from? and (3) How far are they moving?

In the following sections, we describe how established approaches are starting to be combined in novel ways to answer these questions in free‐living (i.e. non‐host associated) environments. Only by quantifying these components of dispersal (i.e. rate, vectors, and distance) can we begin to integrate dispersal into more predictive models and eventually, applications.

### What rate are microbes moving?

Dispersal rate can be measured as the number of individuals immigrating into a defined area per unit time. This measure is analogous to propagule pressure in invasion biology, a measurement describing the number of individuals entering a new habitat per unit time (Simberloff, 2009). In larger organisms, shifts in propagule pressure have been shown to alter establishment success of the invader as well as species composition and diversity in the resident community (Cadotte, 2006; Myers & Harms, 2009; Thomsen et al., 2006). Controlled laboratory studies suggest that the rate of microbial dispersal may also alter the relative importance of dispersal in microbial community assembly whereby high dispersal rates increase the likelihood of establishment in the resident community (Jones et al., 2017). Yet, the rate at which microbes actually disperse in the environment remains largely unknown.

Measuring dispersal rates of microbes poses many technical challenges, but one method that has been employed is the use of microbial ‘traps’, analogous to seed traps used in plant biology (Bullock et al., 2006). For example, in our work, we placed sterile glass slides onto the soil surface to capture the bacterial cells immigrating into the soil surface of a semi‐arid grassland in Southern California. The traps allow one to quantify the number of individuals accumulating over time, while minimizing the possibility of reproduction (Walters et al., 2022). We found that 7900 bacterial cells/cm^2^/day disperse into the soil surface. Similar approaches have been used to quantify dispersal in other systems. For example, Jones et al. (2008) found that 5.7 × 10^8^ bacterial cells/m^2^/h were deposited from the atmosphere into the surface of freshwater lakes in Wisconsin, USA during the first hour of rainfall. Active air samplers are also commonly used to assess aerial dispersal of bacterial and fungal spores (see Egan et al., 2014; Limpert et al., 1999; Prospero et al., 2005), and in aquatic systems, sterile filters could be deployed to assess microbial dispersal in liquid environments at points of interest (e.g. effluent discharge points from wastewater treatment plants, river channels, or ocean currents). Cell abundance on such filters can be measured across time and, in combination with flow rates, be used to quantify natural rates of microbial dispersal within various aquatic settings. Employing such methodology to quantify dispersal rates across different types of ecosystems will help inform more realistic laboratory studies. They will also help to address questions such as: (i) how do dispersal rates vary across landscapes, ecosystems, and time? (ii) how does the rate of dispersal contribute to rates of genetic and ecological drift? and (iii) how does dispersal rate influence microbial community assembly?

### Where are microbes coming from?

At the landscape scale, microbes move passively through the environment via a variety of different dispersal routes. A dispersal route includes both the source community (e.g. soil or sewage treatment plant) and the physical vector (e.g. wind, ocean currents, or an animal host) used to transfer individual cells (Walters et al., 2022). The composition of microbes immigrating through distinct routes can be characterized by sampling each vector independently. For instance, Chaudhary et al. (2020) conducted passive air sampling in the field for 12 months to identify traits of aerially dispersed fungal spores. Similarly, other studies have characterized the composition and diversity of microbes transported through rainwater (Evans et al., 2020), bumblebees (Brysch‐Herzberg, 2004), and snowmelt (Malard & Pearce, 2022). Given that the composition and abundance of microbes transported through each dispersal vector can vary, the effects of dispersal on the resident community functioning may be route specific.

Dispersal exclusion experiments can also be employed to isolate the effects (and rates) of specific dispersal routes. For instance, sequential exclusion of individual dispersal routes has been accomplished by using a series of cages varying in mesh size or orientation. Vannette and Fukami (2017) caged *Mimulus auranticus* flowers using bags with varying mesh sizes to exclude different classes of pollinators, finding that dispersal by hummingbirds but not smaller pollinators influenced the nectar microbiome. Similarly, Walters et al. (2022) manipulated the location and orientation of open litter bags on the soil surface to sequentially exclude dispersal from soil, standing vegetation, and air into senescing leaf litter. In this system, dispersal from air and surrounding vegetation, but not the bulk soil, influenced microbial community assembly.

These studies are rare examples of how dispersal routes can be isolated and assessed in the field and can serve as inspiration for similar assessments in other systems. Among other benefits, identifying important dispersal routes would aid in predicting a microbial community's ability to respond to future global changes (Comte et al., 2017). Further, assessing route‐dependent effects of dispersal may provide a new avenue for characterizing and controlling the spread of pathogens (Gilet & Bourouiba, 2014).

### How far are cells dispersing?

**FIGURE 1:**
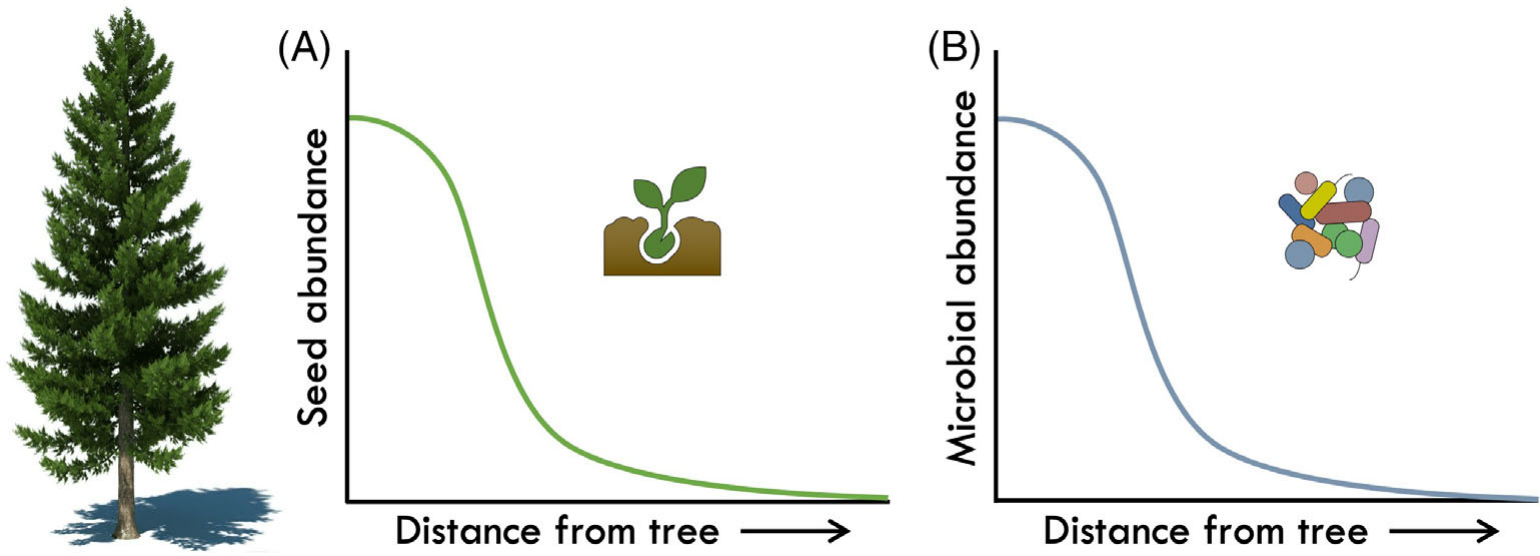
Hypothetical dispersal kernels for (A) plant seeds and (B) microbial communities dispersing away from a tree. When dispersal is limited, the number of propagules decreases with increasing distance from the dispersal source.

Addressing the two previous questions would characterize the way in which microorganisms disperse *into* a resident community of interest. Yet, we also know very little about the distance that microbes disperse *away* from a single source in an environment. Plant ecologists often describe seed dispersal in terms of a dispersal kernel—a probability density function of the likelihood of a single seed dispersing to each distance from a defined source (Nathan & Muller‐Landau, 2000). In a typical example, most seeds will disperse a short distance away from the parent plant with a sharp decrease in the number of seeds dispersing at longer distances (Figure 1A). We propose that microbes will display a similar dispersal pattern (Figure 1B). However, due to technical limitations in tracking (or even defining) a single microbial species, only a handful of dispersal kernels have been previously characterized for microbes. These examples often fall into two categories: (i) pathogens (Gorris et al., 2018; Grosdidier et al., 2018) and (ii) symbionts with narrow host ranges (Peay et al., 2010, 2012).

Albeit challenging, quantifying dispersal distance is necessary to understand the drivers of microbial community assembly across spatial scales. Measuring the dispersal range of most microbes, especially those that are highly abundant in the environment, is not feasible because a single taxon may be dispersing from a variety of different sources. One potential solution is to isolate a particular taxon of interest, develop a trackable strain (e.g. fluorescently labelled), and then follow its spread following reintroduction in the field. Although theoretically possible, this method is not currently practical due to technical limitations in re‐isolating the same strain from the environment. We propose that an alternative, more practical approach to quantifying microbial dispersal is to consider whole community dispersal rates. In this case, the dispersal kernel reflects the probability of any individual from the community emigrating a certain distance from a source of interest (Figure 1B).

**FIGURE 2:**
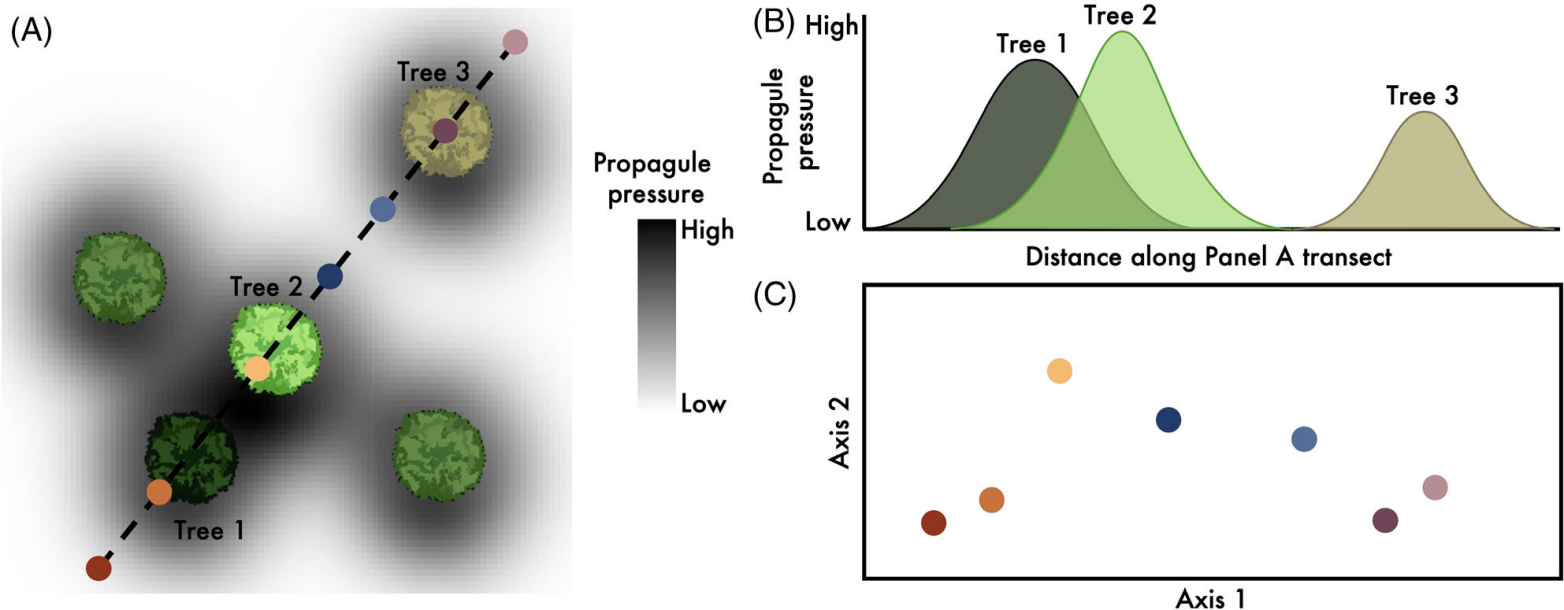
(A) A hypothetical landscape of microorganisms dispersing from multiple sources, here indicated as different colour trees. Tree colour represents unique tree species with distinct dispersal communities (Tree 1–3). Coloured points along a transect (dashed line) indicate the locations of microbial ‘traps’ that are placed on the soil surface to sample the dispersing community. (B) Representative dispersal kernels for three trees along the transect. Each tree has its own unique dispersal kernel. (C) Ordination of the community composition of the microbial trap samples from (A). If dispersal is limited and the sources differ in their microbial composition, then the samples will cluster based on geographic distance to the same dispersal source (trees).

To understand how one might investigate whole microbial community dispersal kernels in the field, consider a hypothetical landscape (Figure 2). In this landscape, microbial traps are deployed at various distances away from key dispersal sources such as standing vegetation (Figure 2A). The traps allow researchers to first quantify dispersal rate (i.e. number of microbes immigrating per unit area per unit time) and their spatial heterogeneity in relation to different potential sources. In addition, if the sources vary in composition, then one can start to consider the overlapping influence of the distinct dispersal kernels (Figure 2B). In this example, the composition of bacteria dispersing from different trees may vary as plant host filtering alters the phyllosphere microbiome. For instance, Meyer et al. (2022) found that initial differences in phyllosphere composition between tomato, pepper, and bean plants led to differential effects on the assembly of microbial communities on the surface of neighbouring tomato plants. Consequently, if dispersal is limited at the landscape scale, then the composition of dispersing individuals would be more similar the closer you sample to a specific plant species (Figure 2C). Thus, by characterizing the number and composition of microbes dispersing across a landscape, we can begin to understand the basic principles of microbial dispersal in the environment and address all three of our questions.

## MOVING FORWARD

Studying the movement of microscopic organisms is undeniably challenging. However, this difficulty should not dissuade us from trying to quantify the basic properties of dispersal (i.e. rate, vectors, and distance) in environmental ecosystems. The studies highlighted above give hints at the future: research in this area will move away from the lab and inference‐based observations and towards field‐based experiments. This will happen not only through technical advances in sequencing, but through clever experimental designs that permit us to measure and manipulate microbial dispersal in the environment. These future studies will yield field‐informed dispersal parameters that can be incorporated into conceptual and predictive models of microbial communities to address far‐reaching questions such as: How do antibiotic‐resistant bacteria or plant pathogens move through a landscape? Can translocation of microbial communities help to restore native plant communities or improve agriculture soils in the face of global change? and Will microbial dispersal allow ecosystems to adapt to future climate change?

## AUTHOR CONTRIBUTIONS

**Kristin Barbour:** Conceptualization (equal); writing – original draft (lead); writing – review and editing (lead). **Alberto Barron Sandoval:** Conceptualization (equal); writing – original draft (supporting); writing – review and editing (supporting). **Kendra E. Walters:** Conceptualization (equal); writing – original draft (supporting); writing – review and editing (supporting). **Jennifer B. H. Martiny:** Conceptualization (equal); supervision (lead); writing – original draft (supporting); writing – review and editing (supporting).

## CONFLICT OF INTEREST

The authors declare no known competing financial interests or personal relationships that could have appeared to influence the work reported in this paper.

## Data Availability

No experimental data was generated or used in this experiment.
